# A Quantitative Index of Sociality and Its Application to Group-Living Spiders and Other Social Organisms

**DOI:** 10.1111/eth.12028

**Published:** 2012-11-19

**Authors:** Leticia Avilés, Gyan Harwood, W Koenig

**Affiliations:** Department of Zoology, University of British ColumbiaVancouver, BC, Canada

## Abstract

Species are often classified in discrete categories, such as solitary, subsocial, social and eusocial based on broad qualitative features of their social systems. Often, however, species fall between categories or species within a category may differ from one another in ways that beg for a quantitative measure of their sociality level. Here, we propose such a quantitative measure in the form of an index that is based on three fundamental features of a social system: (1) the fraction of the life cycle that individuals remain in their social group, (2) the proportion of nests in a population that contain multiple vs. solitary individuals and (3) the proportion of adult members of a group that do not reproduce, but contribute to communal activities. These are measures that should be quantifiable in most social systems, with the first two reflecting the tendencies of individuals to live in groups as a result of philopatry, grouping tendencies and intraspecific tolerance, and the third potentially reflecting the tendencies of individuals to exhibit reproductive altruism. We argue that this index can serve not only as a way of ranking species along a sociality scale, but also as a means of determining how level of sociality correlates with other aspects of the biology of a group of organisms. We illustrate the calculation of this index for the cooperative social spiders and the African mole-rats and use it to analyse how sex ratios and interfemale spacing correlate with level of sociality in spider species in the genus *Anelosimus*.

## Introduction

Sociality is generally understood to be the propensity of individuals to live in groups. Wilson (1975) defines it as the tendency of individuals of the same species to live in groups and display reciprocal, cooperative behaviour. Conventionally, species are placed in discrete categories, such as *solitary*, *subsocial*, *social* and *eusocial*, primarily based on some qualitative features of their natural history, such as generational overlap and the presence or absence of reproductive division of labour (Wilson 1975). While it is certainly convenient to categorize species based on such qualitative features, there is often a gradation in traits related to sociality both among and within categories or even among populations of the same species. Such gradation may be useful to quantify not only as a means of characterizing more subtle differences among species but also as a means of assessing how other behavioural and life-history traits correlate with or are influenced by, how ‘social’ a species is. While continuous indices have been developed that quantify such aspects of sociality as reproductive skew (Sherman et al. 1995; Nonacs 2000), dominance hierarchies (Bang et al. 2009) and social complexity (Blumstein & Armitage 1998), such indices do not account for the most fundamental aspects of group-living, such as philopatry, grouping tendencies and conspecific tolerance.

Here, we propose an index of sociality that is based on three fundamental features of social living: (1) the fraction of their life cycle that group members spend within their social group; (2) the proportion of nests in a population containing multiple vs. solitary individuals; and (3) the proportion of adult members of a group that do not reproduce, but participate in communal activities. The first two components can be taken as reflecting degree of philopatry, grouping tendencies and intraspecific tolerance; and the third as reflecting the tendencies of individuals to exhibit reproductive altruism. We combine these three components in an index that ranges between zero and one, with zero representing completely solitary species and values close to 1.0 representing eusocial species.

The first component of the index can be estimated from the age at which individuals of a given species disperse from their social group relative to the age at which individuals reach adulthood. Individuals that disperse soon after nascence can be considered less social than those that disperse later in their development or those that do not disperse at all. The second component is estimated as the proportion of nests or colonies in a population that contain multiple adult individuals relative to the number of solitary adults and/or breeding pairs in a population. We presume that a higher ratio of multiple adult nests to solitary and breeding pair nests is indicative of greater grouping tendencies and greater tolerance of conspecifics. The third component is estimated as the proportion of adult members of a group that do not reproduce, but contribute to the group's communal activities. Although this third component is akin to indices of reproductive skew (Sherman et al. 1995; Nonacs 2000), such indices can be used more broadly to represent variance in resource distribution among members of a group or population (Kokko et al. 1999), while here we emphasize the fraction of a group's constituents that can be classified as non-reproductive helpers. As such, this component addresses animals that exhibit reproductive division of labour, such as eusocial species, and posits that species with a larger fraction of adult non-reproductive helpers should be considered more social. Taken together, these three measures are quantifiable components of a social system that apply to all species to a greater or lesser extent and so should be sufficient to cover the whole range of social systems in a group of organisms.

To illustrate the calculation of this index, we apply it here to the case of group-living spiders that share a single communal nest in which they remain for part or all of their life cycle (Avilés 1997; Lubin & Bilde 2007) and to the African mole-rats (Bennett & Faulkes 2000). In the case of the spiders, we also illustrate potential uses of the index by analysing how its value, as estimated for species in the spider genus *Anelosimus*, correlates with other aspects of the biology of these organisms, such as sex ratios and interfemale spacing. Other traits that may also correlate with level of sociality in spiders, but are not considered here, include dispersal propensity (Corcobado et al. 2012), degree of inbreeding (Avilés 1997), sexual size dimorphism, the extent to which males participate in communal activities and the degree of allomaternal care (Samuk et al. 2012).

## Materials and Methods

### Study Species

Spiders that live in single communal webs – as opposed to those that aggregate individual territorial webs (Uetz & Hieber 1997) – can be broadly categorized into three discrete categories according to the duration of their social phase: *solitary* species display little or no parental care and offspring disperse at a young age; *subsocial* (or non-territorial periodic social, *sensu* Avilés 1997) species have prolonged periods of parental care and offspring cooperate with one another until they disperse; *social* (or non-territorial permanent social) species have prolonged periods of parental care and adult females cooperatively raise young together, with many offspring forgoing dispersal altogether. However, it is clear that there is a great deal of variation among species that fall into the same discrete category. Such is the case, for instance, for timing of dispersal, among subsocial and solitary species, or in the proportion of colonies containing single individuals, among the social species (Avilés et al. 2007; Lubin & Bilde 2007), with the latter being a trait that may also vary among populations of the same species (Jones et al. 2007; Purcell & Avilés 2007). Additionally, while many species fall cleanly within one of these categories, others appear intermediate in their characteristics (Avilés et al. 2007; Jones et al. 2007).

All spider species used here to illustrate the estimation of the index are from the genus *Anelosimus* (Araneae: Theridiidae) and have been the subject of study to greater or lesser extent by L. Avilés and collaborators starting in the 1980s. Sociality has evolved independently on several occasions in this clade (Agnarsson 2006; Agnarsson et al. 2007) and its species can be found on every continent but Antarctica. The six species included in this paper are all found in Ecuador (Tables 1–3).

**Table 1:** *Anelosimus* spp. of Ecuador and their sociality index scores

Species	Location	Province	Coordinates	Habitat type	Median disperal instar^a^	Proportion of nests with multiple adult females^b^	Proportion non-reproducing adult female potential helpers^c^	Sociality score	Discrete social category
*Anelosimus domingo*	Jatun Sacha	Napo	1.07254 S, 77.61561 W	Lowland rain forest	7	0.96	0.55	0.84	Social
*Anelosimus eximius*	Various (see below)	Various	1.07254 S, 77.61561 W	Lowland rain forest	7	0.74	0.74	0.82	Social
*Anelosimus guacamayos*	Cocodrilos	Napo	0.64928 S; 77.79460 W	Montane cloud forest	7	0.52	0.0 – 0.51	0.51 – 0.68	Social
*Anelosimus baeza*	Yanayacu	Napo	0.60660 S, 77.89469 W	Montane cloud forest	6	0.36	0	0.41	Subsocial
*Anelosimus elegans*	Cocodrilos	Napo	0.64928 S; 77.79460 W	Montane cloud forest	6	0.03	0	0.30	Subsocial
*Anelosimus studiosus*	Calderón	Pichincha	0.00027 S, 78.45474 W	Semiarid	5	0	0	0.24	Subsocial

a Dispersal instar data from the following sources: *A. domingo* (L. Avilés & P. Salazar, unpubl. data, July/August 2001, N = 24 colonies), *A. eximius* (Avilés 1992), *A. guacamayos* (Iturralde 2004), *A. baeza* (G. Corcobado-Marquez, unpubl. data, August 2004, N = 223 newly established nests), *A. elegans* (Iturralde 2004) and *A. studiosus* (L. Avilés, unpubl. data, July/August, 1989, N = 29 newly established nests). All species listed have a total of 7 post-eclosion instars.

b Proportion of nests with multiple adult females based on data from the following sources: *A. domingo* (L. Avilés & P. Salazar, unpubl. data, July/August 2001, N = 24 colonies), *A. eximius* (Purcell & Avilés 2007), *A. guacamayos* (Avilés et al. 2007), *A. baeza* (L. Avilés, unpubl. data, August 1999, N = 1; January 2002, N = 9; June 2005, N = 1 colonies), *A. elegans* (K. Samuk & L. Avilés, unpubl. data, 2009, N = 69 colonies) and *A. studiosus* (L. Avilés, unpubl. data, July/August 1989, N = 102 colonies).

c Proportion of non-reproducing females calculated using data from the following sources: *A. domingo*, Salazar (2001); *A. eximius*, Avilés & Tufiño (1998); *A. guacamayos*, Salazar (2006). Because in the three social species, the proportion of females that reproduce is not constant, but a declining function of colony size, this component of the index was calculated by pooling data from all colonies and dividing the total number of egg sacs produced over a generation by the number of adult females present in the colonies in that generation, excluding solitary females that did not reproduce (both estimated through biweekly censuses of the colonies over one or more generations). *A. guacamayos* low estimate assumes that none of the non-reproducing females help, the higher that all do, bracketing probable sociality scores for this species between 0.51 to 0.68. The lower estimate is unlikely, however, as even if non-reproducing females did not alloparent, they likely participate in web building and prey capture activities.

**Table 2:** *Anelosimus eximius* scores from different localities and *Anelosimus guacamayos* scores from different years

Species	Year^a^	Location	Province	Coordinates	Elevation	Habitat type	Median disperal instar	Proportion of nests with multiple adult females	Proportion of non-reproducing adult female helpers	Sociality score
*Anelosimus eximius*	2007	Cuyabe no (forest & river)	Sucumbios	0.028°S, 76.294°W	225 m	Lowland rain forest	7	0.96	0.71	0.89
*Anelosimus eximius*	2007	Jatun Sacha	Napo	1.07254 S, 77.61561 W	400 m	Lowland rain forest	7	0.91	0.71	0.87
*Anelosimus eximius*	2007	Macas	Marona-Santiago	2.3°S, 78.1°W	1200 m		7	0.76	0.71	0.82
*Anelosimus eximius*	2007	Via a Loreto	Orellana	0.703°S, 77.736°W	1000 m		7	0.64	0.71	0.78
*Anelosimus eximius*	2007	Puyo	Pastaza	1.5°S, 77.9°W	900 m		7	0.39	0.71	0.70
*Anelosimus eximius*	2007	All locations	Various				7	0.74	0.71	0.82
*Anelosimus guacamayos*	2004	Cocodrilos	Napo	0.64928 S; 77.79460 W		Montane cloud forest	7	0.52	0.51	0.68
*Anelosimus guacamayos*	2009	Cocodrilos	Napo	0.64928 S; 77.79460 W		Montane cloud forest	7	0.25	0.51	0.59

a Data from the following sources: *A. eximius* (Purcell & Avilés 2007), *A. guacamayos* [2004] (Iturralde 2004) and *A. guacamayos* [2009] (K. Samuk, unpubl. data, N = 69 colonies). *A. guacamayos* sociality scores may be lower if it is found that non-reproducing females don't help (see Table 1).

**Table 3:** Sex ratio (proportion of males, best available estimate) and nearest neighbour (closest adult female to an adult female) data for six social and subsocial *Anelosimus* species

Species	Proportion males^a^	Instar for sex ratio estimate	Locality	Median nearest neighbour (cm)^b^	Locality
*Anelosimus domingo*	0.10	Embryos	Various	2.1	Jatun Sacha
*Anelosimus eximius*	0.09	Embryos	Pto. Quito, Pichincha	1	Jatun Sacha
*Anelosimus guacamayos*	0.16	Embryos	Cocodrilos	14	Cocodrilos
*Anelosimus baeza*	0.47	Young adults + subadults	Yanayacu	59	Baños
*Anelosimus elegans*	0.30	Subadults	Cocodrilos	58.5	Cocodrilos
*Anelosimus studiosus*	0.48	Embryos	Calderón	58	Baños

a Data from the following sources: *A. domingo* (Avilés et al. 2000), *A. oritoyacu* (Avilés & Purcell, 2011), *A. eximius* (Avilés & Maddison, 1991), *A. guacamayos* (Avilés et al. 2007), *A. baeza* (L. Avilés, unpubl. data [N = 6 completely collected colonies; field notes 1999, 2002, 2005]), *A. elegans* (Iturralde 2004) and *A. studiosus* (Avilés & Maddison, 1991).

b All nearest neighbour data from K. Samuk, unpubl. data collected in 2009.

African mole-rats (family Bathyergidae) are herbivorous, subterranean rodents that display varying degrees of sociality. Some species are strictly solitary, with individuals only cohabiting during the breeding season or when pups are born (Bennett & Jarvis 1988b; Altuna et al. 1991), while other species are social, with individuals living in colonies for much of their lives. Social colonies are founded by a breeding pair and increase in population as new offspring are born. Colony members cooperate in foraging and maintaining the burrow system (Bennett & Faulkes 2000). There are two species of Bathyergidae, the naked mole-rat (*Heterocephalus glaber*) and the Damaraland mole-rat (*Fukomys damarensis*), that display extreme levels of sociality, including reproductive division of labour, overlap of generations and cooperative care of offspring (Jarvis 1981; Jarvis & Bennett 1993). Thus they can be classified as eusocial according to Wilson's (1971) original definition of the term. We include a brief analysis of African mole-rat species in this study to illustrate how the index applies to non-spider systems where parameter data must be collected or inferred in an alternate way. Moreover, because mole-rat species range from solitary to eusocial, we use this group of organisms to show how the four discrete categories of sociality (solitary, subsocial, social and eusocial) segregate into distinct regions of the index, thereby validating it by not violating traditional classification systems.

### Parameter Estimation

The first component the index addresses what proportion of an individual's developmental phase is spent within its natal social group. Systems where individuals do not disperse at all or only disperse after they have reached adulthood are given a value of 1.0. For some species, determining the age at which individuals disperse relative to the age at which they attain adulthood can be difficult, as it may be impractical to monitor individuals from the day they are born until the day they disperse. Conveniently, spiders and other arthropods undergo a series of moults throughout their lifetime and so can be aged according to the instar they are in (Foelix 2010). Thus, *dispersal instar* and *instar at which adulthood is attained* can be used as units of time for determining this component of the index for spiders. Much of the data required for the index are not in the published literature, so we extracted data from our previous fieldwork and also acquired data from our colleagues (Tables 1–3). To infer the dispersal instar for each of the species, we used records on the instar composition of populations of nests that either included all nests present in a given area or a randomly chosen selection of those available. We then noted the instar and sex of individuals occupying newly established nests, as these would have represented recent dispersers. For all but two of the species, we recognized newly established nests after having mapped all existing nests in an area and then noted the subsequent appearance of new nests. In the case of *Anelosimus domingo*, a social species from the lowland rainforest (Rypstra & Tirey 1989), we have not observed such ‘live’ dispersal events despite months of periodic observations of existing nests (P. Salazar, unpubl. data). However, because we have never seen nests containing single individuals of an instar other than adult females, we infer this to be the dispersal instar. For many species, dispersal may not occur at a single instar but instead over a range of instars (Lubin & Robinson 1982; Schneider 1996; Avilés & Gelsey 1998; Powers & Avilés 2003; Li & Kuan 2006; Agnarsson 2006 etc.), so we chose to use the median instar for our index to reduce the influence of outliers that disperse very early or very late and to more accurately reflect the age at which most individuals disperse.

Calculating the proportion of the life cycle that individuals remain in their social group in vertebrate systems requires determining the age at which individuals become adult, which can be complicated, in particular in systems where reproduction is repressed by an alpha individual or pair, as occurs in social and, eusocial mole-rat species (Jarvis 1981; Bennett & Jarvis 1988b; Burda & Kawalika 1993). A possibility is to consider adulthood to be reached when an individual attains the mean body mass of adults in the population, as done by Bennett & Faulkes (2000), who used the Gompertz growth function to estimate the time to adulthood for the mole-rat species included in this study. Dispersal dates can be obtained for solitary species that breed seasonally. For the social and eusocial species, where dispersal only occurs following periods of heavy rains or the death of the reproductives (Sherman et al. 1991), we maximized at 1.0 the first component of the index as new colonies tend to be founded by individuals that are capable of sexual reproduction.

For the calculation of the second component of the index, the proportion of groups vs. solitary individuals in the population, we define a group as having two or more adults of the *same sex*. The reason is that, in many species, males and females may have different social systems, as is the case, for instance, in lions, where females form the basic social unit with a primarily matrilineal structure, while males form separate coalitions that move among female prides (Packer & Pusey 1982). We also exclude breeding pairs from the definition of a social group, as we are interested in social and cooperative interactions that go above and beyond the reproductive partnership. For spiders, we were able to determine the number of nests containing groups of adults and those containing solitary adults or a single breeding pair using the same records as above (Tables 1 and 3). We calculated the index using only data on adult females, as spider societies are clearly female based: adult males are typically only present in the webs prior to egg laying and parental care is performed exclusively by females (Lubin & Bilde 2007). Additionally, in subsocial species, males wander between females’ webs (Klein et al. 2005), while in the social species males can be quite rare as sex ratios are often highly skewed (Avilés 1997; Lubin & Bilde 2007). Thus, as mothers and their offspring form the basic spider social group, using only females allows for a more meaningful comparison between species that disperse from their natal groups and those that remain together permanently. We chose to compare the proportion of *groups of adults* in the population, rather than the *proportion of adults that live in groups* (Jovani & Mavor 2011) because when the species to be compared form relatively large colonies, the estimate of this component of the index would be close to its maximum value, with little separation among species. Using the *proportion of adults that live in groups* would also indirectly include group size as a component of the index, which in our case would be problematic as the third component of the index, which we discuss next, already includes group size in such an indirect way. We thus opted not to count group size twice. For mole-rats, we obtained from the literature colony composition data and counted multiple and single adult female colonies as done for spiders. In the case of the naked mole-rats, the available data did not distinguish between adult and juvenile females, so we conservatively counted colonies containing fewer than 12 females as being ‘solitary female’, because 10 is a common estimate of litter size for this species (Brett et al. 1991).

The third component of the index is akin to indices of reproductive skew (Sherman et al. 1995; Nonacs 2000) and could thus be calculated using one of several existing formulas, as long as they yield estimates ranging between 0 and 1 (see Nonacs 2000; for a review of proposed indices). Here, we use a simplified version of the algorithm proposed by Keller and Reeve (Keller & Vargo 1993; Reeve et al. 1993; Sherman et al. 1995) by considering only the proportion of group members that do not reproduce and assuming equal variance in reproductive output of reproducing individuals. We do this both for practical reasons, as the latter is more difficult to estimate than the former, and because our focus is on the fraction of colony members that could be considered non-reproductive helpers. In the case of the spiders, it can be debated whether individuals in social spider colonies that fail to reproduce should be considered non-reproductive helpers, as absence of universal reproduction likely reflects competition for resources, rather than socially enforced reproductive division of labour (Avilés 1997; Avilés & Tufiño 1998; Bilde et al. 2007; Lubin & Bilde 2007). However, because such non-reproducing individuals participate nevertheless in communal activities, it can be argued that they behave altruistically, and thus, species with a greater fraction of such altruistic helpers, albeit not eusocial in the strict sense of the term (Crespi & Yanega 1995), could nonetheless be considered more social. To calculate this component of the index in spiders, we determined the proportion of females that reproduced by dividing the total number of egg sacs produced during a given generation by the number of females that were adult during the egg-laying period of that generation excluding solitary females that did not reproduce, as these would not have the opportunity to help. By pooling data from all colonies, we accounted for the fact that the fraction of reproducing females in some species declined as a function of colony size (Avilés & Tufiño 1998). For this component of the index, subsocial species were given a value of 0.0. For mole-rat species, we again used the same colony composition records as for the previous component, and, as for spiders, pooled the total number of reproducing females and non-reproducing females to account for any proportional decline as it relates to colony size. For all social species, we counted each colony as having a single reproducing female unless it was explicitly stated that some colonies contained more than one. In the case of naked mole-rats, we used the subset of colonies examined by Brett (1991, tables 4–1 and 4–3) for which he provided an estimate of the proportion of colony members that were adult and female and assumed that those colonies contained a single breeding female.

### The Index

Once we determined dispersal age or instar, the proportion of nests containing multiple adult females and the proportion of non-reproducing adult females, we combined the three factors to give each species a score that ranged between 0 and 1. To estimate this score, we utilize the following formula, which gives equal weight to each of the three components, although different weighting schemes could also be considered:


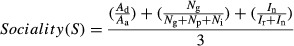
(1)

where *A*_d_ = age at dispersal, *A*_a_ = age when adulthood is reached, *N*_g_ = number of groups, *N*_p_ = number of mating pairs, *N*_i_ = number of solitary adults of the sex whose sociality index is being estimated, *I*_r_ = the number of reproducing adults and *I*_n_ = the number of non-reproducing adults.

We thus estimated the sociality score for six spider and six mole-rat species (Tables 1 and 4). For the case of the spiders, we also collected data from multiple localities for one of the social species, *Anelosimus eximius*, to illustrate regional variation in a species’ level of sociality and from a population of the social *Anelosimus guacamayos* (Avilés et al. 2007) sampled 5 yr apart, to determine consistency of scores taken at different times. With the spider data, we then compared each species’ score against two other life-history and behavioural traits, sex ratio and interfemale spacing, to see what kind of correlation exists between a species’ sociality score and other traits associated with social living. We used our best available estimate of sex ratio, which for most of the species, was the primary (embryonic) sex ratio (Table 3). The interfemale spacing is the median of the distance to the nearest adult female, corrected for body size. Distances were measured on randomly chosen females in a population and could include either another female in the same nest, if the nest contained multiple females, or a female in a nearby nest, if the original female lived alone. The estimate thus reflects the distribution of females in space for species ranging from solitary to fully social. We used the median distance rather than the mean because the median more accurately reflects the precision of our estimates, on the one hand, and the spacing of females in the presence of outliers. For example, if a species generally lives in large colonies where females are closely spaced, then the presence of a single solitary female many metres away will increase the mean spacing to a distance that is not reflective of the conditions facing most individuals in the population. To measure interfemale distance, we drew transects through a population of nests and then chose for measurement nests found nearest the one or five metre mark along the transect, depending on the density of nests in the population. If the nests contained multiple adult females, we then drew imaginary transects through the nests and chose the female closest to every 10-cm mark and then measured the distance to her closest adult female neighbour.

**Table 4:** African mole-rat social data and sociality scores

Species	Common name	Social category	Projected time to attain mean adult body mass^a^	Age at dispersal^b^	Proportion of colonies with multiple adult females^c^	Proportion of non-reproducing adult female potential helpers^d^	Sociality score
*Heterocephalus glaber*	Naked mole-rat	Eusocial	Variable	Adult	0.88	0.96	0.95
*Fukomys damarensis*	Damaraland mole-rat	Eusocial	436 d	Adult	0.50, 0.83	0.90	0.80, 0.91
*Fukomys mechowii*	Giant/Mechow's mole-rat	Social	299 d	Adult	0.72	0.56	0.76
*Georychus capensis*	Cape mole-rat	Solitary	143 d	55–60 d	0	0	0.14
*Bathyergus suillus*	Cape Dune mole-rat	Solitary	227 d	60–65 d	0	0	0.09
*Bathyergus janetta*	Namaqua Dune mole-rat	Solitary	223 d	60–65 d	0	0	0.09

a From Bennett & Faulkes (2000); based on Gompertz growth curves for empirical data. Original sources: Bennett et al. (1991) (*B. suilus, B. janetta, G. capensis*); Bennett & Aguilar (1995) (*F. mechowii*); Jarvis, 1991 (*F. damarensis*); Brett et al. (1991) and O'Riain, 1996 (*H. glaber*).

b Sources: Bennett et al. (1991) (*B. suilus, B. janetta*); Bennett & Jarvis (1988a) (*G. capensis*); Bennett & Aguilar (1995) (*F. mechowii*); Bennett & Jarvis (1988b) and Jarvis & Bennett (1993) (*F. damarensis*); Jarvis, 1991 (*H. glaber*).

^c,d^Sources: Jarvis and Bennett, 1991 (*B. suilus, B. janetta, G. capensis*); Sichilima et al. (2008a,b) (*F. mechowii*); Jarvis & Bennett (1993) (*F. damarensis*); Brett et al. (1991) (*H. glaber*).

## Results

Of the six spider species analysed, three are discretely categorized as social and three as subsocial. Among the social species, dispersal only occurred during the seventh and final instar and the proportion of nests containing multiple adult females ranged from 0.52 to 0.96. Across all colony sizes, the average proportion of adult females not reproducing in the three social species ranged between 0.51 and 0.71. For subsocial species, dispersal occurred in the pre-adult instars (5th and 6th moults) and the proportion of nests containing multiple adult females ranged from 0 to 0.36. In these species, adult females were typically associated with one egg sac (or group of young juveniles) each, including in the few nests that contained more than one female. Thus, the non-reproductive helpers component is 0. This yields sociality scores ranging from 0.68 to 0.84 for social species and 0.24 to 0.41 for subsocial species (Table 1).

While the median dispersal instar remained unchanged for species at different locations or years, this was not the case for the proportion of nests containing multiple adult females. Thus, *A. eximius* populations in areas near the edge of its elevational range had proportions between 0.39 and 0.76 of nests with multiple females, compared with proportions of over 0.9 at the lower elevations (Purcell & Avilés 2007). This resulted in sociality scores between 0.70 and 0.82, at the higher elevations, and between 0.87 and 0.89 at the lower ones (Table 3). Likewise, an *A. guacamayos* population surveyed 5 yr apart had a twofold difference in the proportion of multifemale nests between years and sociality scores of 0.59 to 0.68.

**Fig. 1:**
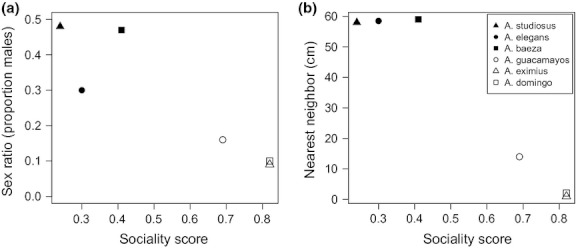
(a) Sex ratio (proportion of males among developing embryos or earliest developmental stage for which data were available) and (b) nearest neighbour distance (distance between adult females) for six *Anelosimus* species of various levels of sociality, respectively, plotted against their estimated sociality score. Filled symbols denote subsocial species, and unfilled symbols denote social species.

The nearest neighbour and sex ratio data (Table 3) were plotted against each species’ sociality score. As expected, species with higher sociality scores tended to have more skewed sex ratios (Fig. 1a). Median nearest neighbour distances were short among social species, which was expected as most nearest neighbours were found within the same nest. Nonetheless, the midelevation *A. guacamayos* exhibited greater spacing among females than the two social species in the lowland rainforest (Fig. 1b). Surprisingly, however, all three subsocial species also had very similar spacing between females, even though there was no a priori reason to expect this to be the case; social and subsocial species were thus separated into very distinct regions on the regression plot.

African mole-rats show a wide range of social structures, which is consistent with the range of sociality scores received by the various species. The three solitary species examined do not live in groups and hence also do not possess non-reproducing helpers, so their low scores (0.09–0.14) are determined strictly by the proportion of their lives that they spend in their natal burrow prior to dispersal. In the social species, *Fukomys mechowii*, approximately 72% of colonies contain multiple adult females and around 56% of adult females in the population do not reproduce in a given year (Sichilima et al. 2008a,b), resulting in a sociality score of 0.76. The eusocial *Fukomys damarensis* disperse as adults and about 90% of individuals live a lifetime of socially induced sterility (Jarvis & Bennett 1993), but heavy rains can sway individuals to disperse and establish new colonies. Thus, the proportion of colonies containing multiple vs. single adult females ranged from 0.50 in one year to 0.83 in another, resulting in sociality scores of 0.80 and 0.91, respectively. The other eusocial species, *Hetercephalus glaber*, also disperses as adults and approximately 96% of adults are non-reproductive. We conservatively estimated the proportion of multiple adult female colonies vs. single adult female colonies as 0.88, which resulted in a sociality score of 0.95, but this score is likely to be even higher. Table 4 above is meant to illustrate how the sociality index can apply to non-arachnid systems and systems that include eusocial species and is not intended to be a rigorous analysis of mole-rat sociality.

## Discussion

The sociality index we have developed is both consistent with and supports traditional classification systems, as species from the same discrete social category inhabit a similar region in the range of possible scores. Solitary mole-rat species receive the lowest scores, ranging between 0.09 and 0.14, which likely would also be the case for solitary spider species given the early timing of their dispersal, the total lack of nests containing more than one adult female and the absence of non-reproducing helpers. Subsocial spiders receive scores between 0.24 and 0.41, while social spiders receive scores between 0.59 and 0.84. The social mole-rat, *Fukomys mechowii*, receives a score of 0.76, which is comparable to scores of social spider species, while the two eusocial mole-rats receive the highest scores at 0.91 for *Fukomys damarensis* and 0.95 for *Hetercephalus glaber*, consistent with the expectation of eusocial species having the highest level of sociality. The one area of slight overlap is for *Fukomys damarensis*, which, after a year of heavy rains, increased the frequency of newly founded, single-female colonies, with a concomitant drop of its sociality score to 0.80 (Table 4). That sociality scores may vary with correlated environmental changes, however, is a strength rather than a weakness of the proposed index.

In addition to being consistent with discrete categorizations of sociality, our quantitative index displays sufficiently large differences among species of the same category or among populations of the same species, to make its development worthwhile. This is nicely illustrated, for instance, with the scores received by different populations of *A. eximius* (Table 2), which are consistent with the observation that populations towards the edge of the elevational range limit of this species are less social than those at the core (Purcell & Avilés 2007). Likewise, previous studies have suggested that *A. guacamayos*, a higher elevation social species, exhibits a level of sociality intermediate between subsocial and social, consistent with the somewhat intermediate index obtained for this species (Table 2). A declining level of sociality with elevation in spiders is thought to reflect an underlying decline in the size of insects available as prey (Guevara & Avilés 2007; Powers & Avilés 2007; Yip et al. 2008). A numerical index as the one we propose would thus also allow us to quantify the extent to which level of sociality correlates with particular abiotic or biotic factors.

The data on *A. guacamayos* spiders and *F. damarensis* mole-rats show that estimates taken at different times may not be identical, either due to measurement error or, more likely, because the size and frequency of single-female nests may vary from year to year or with the season. Social mole-rats and Ecuadorian social *Anelosimus* species are aseasonal breeders, so the frequency of single-female nests should not vary much throughout the year. However, for seasonal breeders, any synchronized dispersal can result in a large increase in the number of new breeding pairs and solitary adults in the population at particular times of the year, which will in turn result in scores varying at different times of the year. Therefore, it is important to collect data at key points of the life cycle, such as towards the end of the species’ dispersal period, when the tendency of individuals to form group vs. nest alone can be best appraised and halfway during the egg-rearing period when the degree of communal brood care can best be ascertained. In many species, dispersal is sex specific, which may cause disparities in the sociality scores of males and females. Thus, it is critical to keep records for both males and females so that such disparities can be recognized and cross-species comparison performed accordingly.

Because the index takes into account the proportion of non-reproducing helpers, group size invariably becomes a discerning factor between species with some degree of reproductive skew, as large, queen-based societies would have the highest ratio of non-reproductive to reproductive individuals. Nonetheless, group size only has an indirect influence on the index, and only when comparing among species that would score above 0.66. While some may feel that group size is a key factor in determining how social a species is and should thus be included as its own component of the index, it can be argued that colony size may be influenced by elements not related to a species’ social behaviour, such as body size, clutch size and food availability. Hence, species that live in small, highly cohesive social groups may be unfairly scored as being less social than others under such a scoring regime.

Spiders with similar sociality scores showed similarity in behavioural and life-history traits that correlated with sociality. Thus, as shown in Fig. 1, more social species tend to have more skewed sex ratios, which would be expected if greater sociality in spiders were associated with greater incentives to remain in the natal group and avoid dispersal for both males and females, thereby resulting in increasingly inbred and isolated colony lineages in the more social species, while the subsocial ones remain primarily outbred (Avilés 1993a; Avilés & Purcell 2012). While the sex ratio data show a somewhat gradual increase in degree of female bias with increasing sociality, the nearest neighbour data show a more dramatic distinction between social and subsocial species (Fig. 1b). Although we expected the social species to have similar nearest neighbour distances because the majority of measurements would be between females in the same nests, this was not the case for the subsocial ones where we expected nearest neighbour distances to be much more idiosyncratic. It is unclear whether the similarity among subsocial species reflects a common behavioural phenotype (i.e. similar dispersal distances) or whether it is simply a biproduct of the distribution of adequate nesting sites on similar plant substrates. If it is due to a common dispersal distance, then social and subsocial species would appear to correspond to two discrete behavioural syndromes, whereby a shift from one state to the other requires a qualitative change to the system itself, consistent with a discrete categorization of ‘solitary’, ‘subsocial’ and ‘social’. The latter suggestion is only partially consistent with the findings of Corcobado et al. (2012), who show that there is a significant difference in dispersal tendencies and abilities of social and subsocial spiders, but enough of a gradation within the two categories for the change to be more or less continuous rather than abrupt.

In summary, the index we have proposed not only allows the ranking of species along a sociality scale from solitary to eusocial, but is also useful for quantifying differences among species within and across categories and among populations of a given species. Because it is based on such fundamental aspects of a social system, such as the tendencies of individuals to be grouped, remain in their social groups and act as non-reproductive helpers, it can be extremely general in its applicability. Thus, to the extent that the forces shaping social systems are common across systems and levels of organization, the index can potentially be applied for comparisons not only within (Tables 1 and 4), but possibly also across different groups of organisms. As illustrated here, a quantitative index of sociality can then be used to ascertain how other aspects of the biology of a group of organisms and the environments in which they live may correlate with a sense of how ‘social’ a species is.
